# Neurodevelopmental and genetic findings in neonates with intracranial arteriovenous shunts: A case series

**DOI:** 10.3389/fped.2023.1111527

**Published:** 2023-03-29

**Authors:** Francesca Campi, Domenico Umberto De Rose, Flaminia Pugnaloni, Sara Ronci, Monica Calì, Stefano Pro, Daniela Longo, Giulia Lucignani, Laura Raho, Elisa Pisaneschi, Maria Cristina Digilio, Immacolata Savarese, Iliana Bersani, Paolina Giuseppina Amante, Marta Conti, Paola De Liso, Irma Capolupo, Annabella Braguglia, Carlo Gandolfo, Andrea Dotta

**Affiliations:** ^1^ Neonatal Intensive Care Unit, “Bambino Gesù” Children’s Hospital IRCCS, Rome, Italy; ^2^ Development Neurology Unit, “Bambino Gesù” Children’s Hospital IRCCS, Rome, Italy; ^3^ Neuroradiology Unit, “Bambino Gesù” Children’s Hospital IRCCS, Rome, Italy; ^4^ Clinical Psychology Unit, “Bambino Gesù” Children’s Hospital IRCCS, Rome, Italy; ^5^ Translational Cytogenomics Research Unit, “Bambino Gesù” Children’s Hospital IRCCS, Rome, Italy; ^6^ Genetics and Rare Diseases Research Unit, “Bambino Gesù” Children’s Hospital IRCCS, Rome, Italy; ^7^ Neurosurgery Unit, “Bambino Gesù” Children’s Hospital IRCCS, Rome, Italy; ^8^ Clinical and Experimental Neurology Unit, “Bambino Gesù” Children’s Hospital IRCCS, Rome, Italy; ^9^ Neonatal Sub-Intensive Care Unit and Follow-up, “Bambino Gesù” Children’s Hospital IRCCS, Rome, Italy

**Keywords:** vein of galen malformations, arteriovenous malformation, brain injury, patient outcome assessment, neurodevelopmental disorders, vascular malformations

## Abstract

**Background:**

Despite the latest advances in prenatal diagnosis and postnatal embolization procedures, intracranial arteriovenous shunts (AVSs) are still associated with high mortality and morbidity rates. Our aim was to evaluate the presentation and clinical course, the neurodevelopmental outcome, and the genetic findings of neonates with AVSs.

**Methods:**

In this retrospective observational study, medical records of neonates with cerebral AVSs admitted to our hospital from January 2020 to July 2022 were revised. In particular, we evaluated neuroimaging characteristics, endovascular treatment, neurophysiological features, neurodevelopmental outcomes, and genetic findings.

**Results:**

We described the characteristics of 11 patients with AVSs. Ten infants (90.9%) required embolization during the first three months of life. In 5/9 infants, pathological electroencephalography findings were observed; of them, two patients presented seizures. Eight patients performed Median Nerve Somatosensory Evoked Potentials (MN-SEPs): of them, six had an impaired response. We found normal responses at Visual Evoked Potentials and Brainstem Auditory Evoked Potentials. Eight patients survived (72.7%) and were enrolled in our multidisciplinary follow-up program. Of them, 7/8 completed the Bayley-III Scales at 6 months of corrected age: none of them had cognitive and language delays; conversely, a patient had a moderate delay on the Motor scale. The remaining survivor patient developed cerebral palsy and could not undergo Bayley-III evaluation because of the severe psychomotor delay. From the genetic point of view, we found a novel pathogenic variant in the NOTCH3 gene and three additional genomic defects of uncertain pathogenicity.

**Conclusion:**

We propose SEPs as an ancillary test to discern the most vulnerable infants at the bedside, particularly to identify possible future motor impairment in follow-up. The early identification of a cognitive or motor delay is critical to intervene with personalized rehabilitation treatment and minimize future impairment promptly. Furthermore, the correct interpretation of identified genetic variants could provide useful information, but further studies are needed to investigate the role of these variants in the pathogenesis of AVSs.

## 1. Introduction

Intracranial arteriovenous shunts (AVSs) are rare congenital cerebral anomalies that include pial arteriovenous malformations (AVMs), vein of Galen aneurysmal malformations (VGAMs), and dural sinus malformations (DSMs, in particular dural arteriovenous dural fistulae—DAVF subtype) (1). In particular, VGAM is characterized by an arteriovenous shunting of the arterial limbic system (e.g., choroidal and pericallosal arteries) draining into the vein of Galen forerunner (2). Neonatal presentation of the deriving aneurysmal formation is usually associated with high-output heart failure (3), and later high mortality and morbidity rates, such as seizures and neurodevelopmental impairment, despite the advances in prenatal diagnosis and postnatal embolization procedures (4–7). Furthermore, an accurate neurophysiological and neuroimaging assessment could be useful in predicting the prognosis and improving short- and long-term outcomes. Our aim was to evaluate the presentation and clinical course, neurodevelopmental outcome, and genetic findings of neonates with AVSs.

## 2. Methods

### 2.1. Study design

In this retrospective observational study, medical records of patients with cerebral AVSs admitted to our Neonatal Intensive Care Unit (NICU) and Neonatal Sub-Intensive Care Unit (NSICU) from January 2020 to July 2022 were revised. After discharge, all AVSs survivors are offered to join our dedicated multidisciplinary outpatient follow-up program. Medical history and clinical data concerning NICU hospitalization and follow-up were collected from the electronic medical records.

### 2.2. Neuroimaging and endovascular treatment

Cranial Doppler ultrasound (cUS) was initially performed on the first day of life to confirm the presence of AVSs and depict the initial set-up in all infants, by a trained neuroradiologist, with a convex neonatal probe (multifrequency 5–10 MHz). Afterward, all infants underwent brain magnetic resonance imaging (MRI) within the first 48 h of life. All images were acquired on a 3T Siemens Magnetom Skyra scanner (Siemens, Erlangen, Germany) equipped with 32 channels head-coil (coil dimensions L–W–H: 440 mm × 330 mm × 370 mm). MRI imaging protocol included: sagittal 3D MPRAGE (TR/TE = 2,060/2.2 ms, FA = 9°, ST = 0.8 mm), sagittal SPACE-T2 (TR/TE = 3,000/400 ms, FA = 120°, ST = 0.9 mm), axial, coronal and sagittal TSE T2-weighted sequence (TR/TE = 6,380/108 ms, FA = 150°,ST = 3 mm), axial susceptibility weighted imaging (SWI) sequence (TR/TE = 27/20 ms, FA = 15°, ST = 1.5 mm), axial diffusion-weighted imaging (DWI) (TR/TE = 9,000/98 ms, FA = 90°, ST = 3 mm) and MRA-TOF (TR/TE = 25/3.81 ms, FA = 18°, ST = 1.0 mm).

All patients were clinically assessed by a multidisciplinary team and underwent brain MRI in order to depict contraindications to endovascular treatment (severe brain injuries up to melting brain, secondary to stealing flow or previous extensive hemorrhage or stroke), hemodynamic features (malformation flow balance between input overload and output incompetence), and associated complications (hydrocephalus, aqueduct compression, venous hypertension, etc.). In patients without sharp complications, the Bicêtre Neonatal Evaluation Score (BNES) application eased to decide whether immediate or delayed endovascular treatment was the best option. When eligible for treatment, neonates underwent endovascular treatment by catheterizing internal carotid and vertebral arteries, *via* the femoral artery, with a diagnostic catheter (4 F). Direct high-flow fistulas were selectively and coaxially catheterized using specific microcatheters (distal outer diameter ranging from 1.2 to 1.5 F). The elective embolization method to close the shunts was a super selective transarterial injection of N-butyl-cyanoacrylate (NBCA) in intracranial distal feeding pedicles, occasionally in conjunction with coil embolization. Different glue concentrations were injected under continuous fluoroscopic guidance depending on the AV flow, with a less diluted NBCA-Lipiodol mixture in the event of greater flow rates to minimize the danger of glue migration onto the vein. According to the size of the aneurysm, the initial procedure's aim was to minimize flow-shunt in order to reduce or halt the onset of heart failure.

After the procedure, a day-1 post-operatory computed tomography scan was mandatory to exclude acute ischemic or hemorrhagic complications, while stricter MRI scan follow-up (1-3-6 post-operatory months) guided to plan other treatments when needed.

### 2.3. Neurophysiological features

In our NICU, all infants with intracranial AVs are monitored with amplitude-integrated electroencephalogram (aEEG) in the first 48–72 h. Video-electroencephalogram (EEG) recordings were performed using the 10–20 international system for scalp electrode placement. Electrocardiogram (EKG), pneumogram, and electromyogram (usually two deltoids) were also recorded (8). Furthermore, standard non-invasive neurophysiological tests were performed, assessing the functional integrity of deep brain structures and cortical areas which can be selectively vulnerable in these patients.

Visual Evoked Potentials (VEPs) responses were recorded using a stimulus consisting of white flashes; rate 0.5 Hz, filters 1–200 Hz, analysis time 1,000 ms, delivered monocularly. The montage was Oz with Fz reference. The P200 component was defined as the large positive wave at around 200 ms. Median Nerve Somatosensory Evoked Potentials (MN-SEPs) responses were elicited by electrical stimuli of the median nerve at the right and left wrist, with intensity at the motor threshold, rate 0.5 Hz, duration 0.2 ms, were used. The analysis time was 100 ms. The montage for the N13 component was the 7th cervical vertebra spinous process referred to Fz, for the N20/P25 cortical component was the contralateral central site (C3 or C4) referred to the ipsilateral one (10–20 international system) (9).

To evaluate Brainstem Auditory Evoked Potentials (BAEPs), surface electrodes were placed at the vertex (Cz) and on each mastoid side. The channel derivations included ipsilateral mastoid to vertex, contralateral mastoid to vertex and ipsilateral to contralateral mastoids. For stimulation, clicks with rarefaction polarity were used, at a temporal rate of 11 Hz and intensity of 95 dBnHL. The low band pass filter was 20 Hz, and the high band pass filter 3,000 Hz. The post-acquisition traces were not digitally filtered. Average sweeps between 1,000 and 2,000 were used for each trace, and at least two traces were recorded for each ear (10).

### 2.4. Neurodevelopmental outcomes

Cognitive, motor, and language domains were assessed at 6 months of life using the Bayley Scales of Infant and Toddler Development—3rd Edition (BSID-III).

The cognitive scale is composed of non-verbal tasks involving memory, problem-solving, and manipulation. The motor scale is made up of the fine motor subtest, which assesses visual-motor integration, visual-spatial awareness, and hand-motor control, and the gross motor subtest, which assesses large-body complex movements and mobility. The language scale estimates both receptive and expressive communication, including verbal understanding, concept development, and communication through words and gestures.

Age-standardized scores for each domain were calculated using test norms (100 ± 15). Cognitive, motor, and language outcomes were classified as normal when the obtained score was >85; borderline when from 70 to 84; delayed when was less than 70 (11).

### 2.5. Genetic analysis

Genomic DNA was extracted from circulating leukocytes collected from the probands. Standard karyotype and Single Nucleotide Polymorphism array (SNP-array, with CytoSNP-850K platform at an average resolution of 100 kb) were used to identify chromosomal anomalies or rearrangements. Next-generation sequencing (NGS) was performed, using Twist Custom Panel kit—Clinical Exome (Twist Bioscience), in the platform NovaSeq6000 (Illumina). In silico analysis in trios (proband and parents) was performed for coding regions and exon-intron junctions of the genes associated with vascular malformations: AGGF1, AKT1, PIK3CA, PTEN, SEC23B, STAMBP, GNAQ, GLMN, RASA1, EPHB4, TEK, GNA3, GNA11, GNA14, FOS, FOSB, FLT4, GJC2, VEGFC, CCBE1, ADAMTS3, FAT4, CALCRL, FOXC2, GATA2, SOX18, KIF11, PTPN14, PIEZO1, ELMO2, KRIT1, CCM2, PDCD10, EDN1, PLCB4, HTRA1, COL4A1, COL4A2, NOTCH3, and NOTCH4.

### 2.6. Ethical approval and statistical analysis

The study was approved by our institutional review board, which waived the need for parental consent because of the retrospective design of the study. Furthermore, neurologic assessment and follow-up were part of the standard of patient care. All parents signed a written informed consent to perform genetic analyses on their children, providing the possibility to report eventual genetic mutations in scientific reports.

Data are presented as numbers and percentages for categorical variables. Continuous variables are expressed as mean ± standard deviation (SD) if they were normally distributed or as median and interquartile range if normality could not be accepted, according to the D'Agostino-Pearson test. Data were analyzed with the MedCalc Software package for Windows, release 12.7 (MedCalc Software, Belgium).

## 3. Results

### 3.1. Patients

During the study period, eleven patients with AVSs were admitted to our department. Ten patients (P1–P7, P9–P11: 90.9%) had a VGAM (choroidal type), and a patient (P8: 9.1%) had a DSM (neonatal DAVF subtype) of the posterior cranial fossa.

Neonatal characteristics are summarized in Table 1. Ten patients (90.9%) previously received a prenatal diagnosis, and 45.5% had signs of fetal heart failure.

**Table 1:** Neonatal characteristics.

Neonatal Characteristics	Intracranial AVSs (*n* = 11)
Males, number (%)	8 (72.7%)
Gestational age, weeks	37 ± 2
Apgar score at 5 min	8 (7–9)
Outborn infants, number (%)	6 (55.5%)
Prenatal diagnosis, number (%)	10 (90.9%)
Prenatal heart failure, number (%)	5 (45.5%)
Preterm birth, number (%)	4 (36.4%)

Invasive mechanical ventilation was needed by eight patients (72.7% of cases), whereas nine patients (81.8%) experienced early postnatal cardiac failure requiring inotropic support. The median length of hospital stay was 27 days (IQR 15–65), and 3/11 (27.3%) died due to progressive multiorgan failure. Survivors (8/11, 72.7%) were enrolled in our multidisciplinary follow-up program. Ten infants (90.9%) required embolization during the first three months of life. Currently, only one patient in our cohort has not yet needed an endovascular procedure (at 6 months of life), and he is regularly followed up in our center.

**Figure 1:**
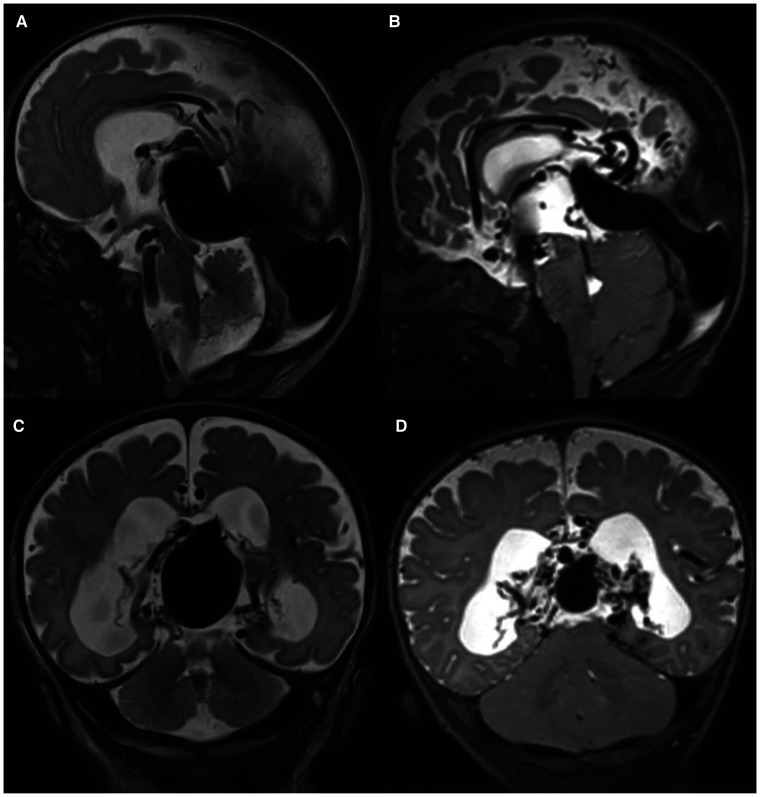
Brain MRI features: T2w sagittal (**A,B**) and coronal (**C,D**) images show the presence of VGAM at birth (**A,C**) and after embolization (**B,D**) in patient P5.

**Figure 2:**
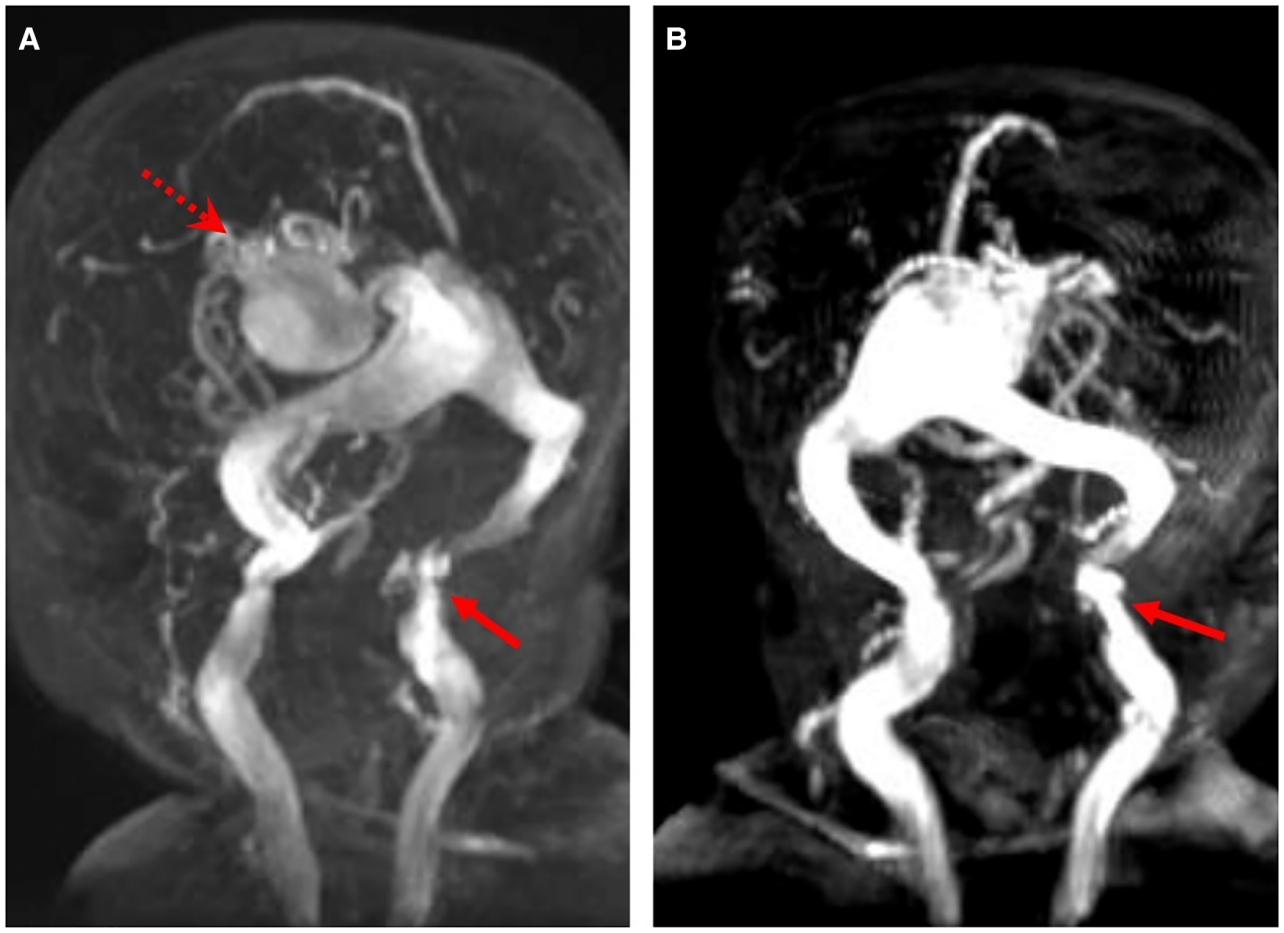
Magnetic resonance angiogram images show the VGAM (red dotted arrow) and the stenosis of the left jugular bulb (red arrow) (**A,B**) in patient P1.

Initial brain MRI characteristics of intracranial AVSs are reported in Table 2: an example of changes before and after embolization was reported in Figure 1, while Figure 2 describes an example of jugular bulb stenosis.

**Table 2:** Outcome, Bicêtre neonatal evaluation score, neuroimaging findings, median nerve somatosensory evoked potentials (MN-SEPs), electroencephalogram findings, genetic findings, and Bayley-III scores.

	Outcome	Bicêtre neonatal evaluative score	MRI findings	MN-SEPs	EEG	Genetic findings	Bayley scores
P1	Survivor	7	VGAM-CT, LJBS	Absent bilaterally	DEA; W-S	–	Cog: 90; Mot: 73; Lang: 103
P2	Survivor	12	VGAM-CT	Absent monolaterally	W-S	–	Cog: 95; Mot: 91; Lang: 103
P3	Survivor	14	VGAM-CT, RJBS	NP	NP	–	Cog: 115; Mot: 91; Lang: 106
P4	Died	3	VGAM-CT, BA	Absent monolaterally	DEA; DSe; FSe; BS.	–	–
P5	Survivor	9	VGAM-CT, Hyd, BA	Absent monolaterally	SO	Microduplication involving the region 11q25, with an extension of 165 kb (131929631_132094964)	Cog: 90; Mot: 91; Lang: 94
P6	Survivor	13	VGAM-CT	Normal	SO	Microduplication of the Xp22.33 region (not pseudoautosomal) of the short arm of the X chromosome (about 195 kb in length, 2782116_2976786)	Cog: 115; Mot: 112; Lang: 94
P7	Survivor	7	VGAM-CT, BJBS	Absent monolaterally	DEA; FSe	Heterozygous c.2903C>A variant in the NOTCH3 gene, with the introduction of the premature stop codon p.Ser968Ter.	Cerebral palsy
P8	Died	4	DSM, VC	NP	DEA; BS	–	–
P9	Died	7	VGAM-CT, Hyd, BA	Absent monolaterally	DEA	–	–
P10	Survivor	12	VGAM-CT	Normal	W-S; SO	Heterozygous c.4855C>A variant in the NOTCH4 gene, determining the amino acid exchange p.Leu1619Met	Cog: 100; Mot: 91; Lang: 94
P11	Survivor	18	VGAM-CT	NP	NP	–	Cog: 105; Mot: 100; Lang: 112

**Neuroimaging findings:** BA, brain atrophy; BJBS, bilateral jugular bulb stenosis; VGAM-CT, vein of Galen aneurysmal malformation—choroidal type; DSM, dural sinus malformation; Hyd, hydrocephalus; LJBS, left jugular bulb stenosis; NP, not performed; RJBS, right jugular bulb stenosis; VC, venous congestion.

**Electroencephalogram (EEG) findings:** BS, burst suppression; DEA, discontinuous electrical activity; DSe, diffuse seizures; FSe, focal seizures; NP, not performed; SO, sleep organization; W-S, wake-sleep alternation.

**Genetic findings:** NP, not performed.

**Bayley scores:** Cog, cognitive; Mot, motor; Lang, language.

The median age at first embolization was 10 days (IQR 6–28). Six infants (54.5%: P2, P3, P6, P8, P9, and P10) required only one procedure, whereas two (18.2%: P1 and P5) needed two embolizations and two (18.2%: P4 and P7) needed three embolizations.

Two infants (P5 and P9) had hydrocephalus and brain atrophy not requiring treatment (already present at birth); one infant (P4) had brain atrophy, already present at birth. One patient (P6) initially had a normal size of ventricles and then developed a mild ventriculomegaly, not requiring treatment during follow-up. Conversely, patient P7 had an intraventricular hemorrhage at 40 days of life (about 30 days after the first endovascular treatment) and then hydrocephalus. An External Ventricular Drain was first inserted, and then a ventriculoperitoneal shunt was placed. Patient P8 was the only one with a DSM (neonatal DAVF subtype) of the posterior cranial fossa, with widespread phenomena of venous congestion.

### 3.2. Neurophysiological features

Nine patients underwent video-EEG during their NICU stay. In five infants (55.6%: P1, P4, P7, P8, P9), pathological EEG findings were observed (Table 2). Two patients (P4 and P8) presented a burst-suppression pattern on the initial EEG. Two patients (P4 and P7) presented electrographic-only seizures, identified at 27 days of life and at about three months of life. Afterward, patient P7 presented focal motor seizures too. In particular, patient P4 presented a low amplitude background activity in the right hemisphere and discontinuous background activity in the left hemisphere, with left occipital and temporal spikes and slow waves; patient P7 presented a low amplitude background activity, mainly in the right hemisphere, in the presence of right temporal slow and sharp waves.

Three patients (1 died and 2 survivors) did not perform evoked potentials. Six patients underwent VEPs with normal responses. Eight patients performed MN-SEPs: one patient with N20 absent bilaterally (survivor), 5 patients with N20 preserved monolaterally (2 died and 3 survivors), and 2 patients with N20 bilaterally normal (both survivors). Three patients underwent BAEPs with normal responses (Table 3).

**Table 3:** Details of neurophysiological features.

	VEPs	MN-SEPs	BAEPs
**P1** (EPs performed at 2 months of age)	N/N	A/A	N/N
	Lat (ms) 148.5/146.7		IPL I–V (ms) 5.05/5.07
	Amp (µV) 13.3/14.2		Amp V wave (µV) 0.18/0.22
**P2** (EPs performed at 1 month of age)	N/N	N/A	NP
	Lat (ms) 173.1/180.5	Lat (ms) 28.5/–	
	Amp (µV) 10.4/12.1	Amp (µV) 2.4/–	
**P3** (EPs performed at 1 month of age)	N/N	NP	NP
	Lat (ms) 180.4/179.4		
	Amp (µV) 12.2/12.9		
**P4** (EPs performed at 1 month of age)	NP	N/A	NP
		Lat (ms) 31.5/–	
		Amp (µV) 2.3/–	
**P5** (EPs performed at 1 month of age)	NP	N/A	N/N
		Lat (ms) 28.2/–	IPL I–V (ms) 4.87/4.77
		Amp (µV) 2.1/–	Amp V wave (vV) 0.23/0.20
**P6** (EPs performed at 1 month of age)	N/N	N/N	NP
	Lat (ms) 169.4/171.4	Lat (ms) 29.5/28.4	
	Amp (µV) 18.1/16.4	Amp (µV) 3.5/3.3	
**P7** (EPs performed at 6 months of age)	N/N	N/A	N/N
	Lat (ms) 132.1/132.6	Lat (ms) 22.1/–	IPL I–V (ms) 4.55/4.43
	Amp (µV) 21.0/22.1	Amp (µV) 3.5/–	Amp V wave (vV) 0.34/0.29
**P8**	NP	NP	NP
**P9** (EPs performed at 2 months of age)	NP	N/A	NP
		Lat (ms) 27.1/–	
		Amp (µV) 2.9/–	
**P10** (EPs performed at 1 month of age)	N/N	N/N	NP
	Lat (ms) 186.6/183.9	Lat (ms) 28.0/29.1	
	Amp (µV) 10.0/11.3	Amp (µV) 2.0/2.3	
**P11**	NP	NP	NP

A, absent; N, normal; NP, not performed. We reported latency and amplitude of VEPs (P200) cortical responses, MN-SEPs (N20/P25) cortical responses, I–V IPL (interpeak latencies) and amplitude V wave of BAEPs.

### 3.3. Neurodevelopmental outcomes

At the time of writing, 7/8 survivor patients enrolled in the present study completed the Bayley-III Scales at 6 months of corrected age (CA). Patient P7 was not evaluated using Bayley-III because of severe psychomotor delay and cerebral palsy.

**Figure 3:**
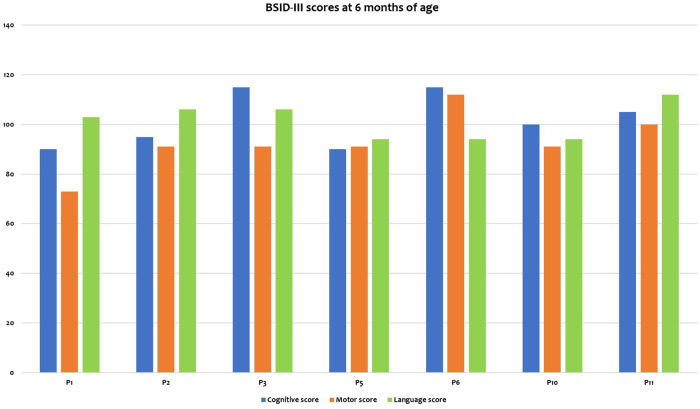
Short-term neurodevelopmental outcomes at 6 months of age.

Among the 7 evaluated patients, the mean scores on the Bayley-III Cognitive, Motor and Language scales were 101.4 ± 10.7, 92.7 ± 11.7, and 101.0 ± 7.3, respectively. Obtained scores are reported in Figure 3. On the Cognitive and Language scales, none of the patients had mild/moderate delay using test norms. Conversely, on the Motor scale, only patient P1 (20%) had a moderate delay (score = 73), with poorly represented spontaneous motor skills, stereotyped limb movements, and reduced motor initiative. The remaining patients showed normal motor scores using test norms.

### 3.4. Relationship between neurophysiology and neurodevelopment

Neurophysiological data collected are few and inhomogeneous; therefore, it is difficult to draw conclusions with clinical neurodevelopmental data.

Five patients performed MN-SEPs and Bayley Scales of Infant Development at 6 months. All five patients had normal cognitive and language scores. Concerning motor skills, one patient without N20 bilaterally had a low score in fine and gross motor skills, whereas the other two patients with N20 absent monolaterally and two patients who had N20 bilaterally preserved had low scores in fine motor skills. In this small group of patients VEPs and BAEPs did not show significant results.

Furthermore, patient P7, who initially had N20 absent monolaterally, had cerebral palsy and could not undergo Bayley-III evaluation because of the severe psychomotor delay.

### 3.5. Genetic findings

Genetic analyses were performed on eight patients, and genetic variants were detected in four cases (50%). The clinical phenotype was normal in all four patients in the first months of life.

In the first case (patient P5), SNP-array showed a microduplication involving the region 11q25, with an extension of 165 kb (131929631_132094964), including the genes NTM and segregating from the mother.

In the second case (patient P6), SNP-array showed a microduplication of the Xp22.33 region (not pseudoautosomal) of the short arm of the X chromosome (about 195 kb in length, 2782116_2976786) and including the genes ARSE, XG, GYG2, and ARSD. At the time of writing, these microduplications are not currently associated with known syndromic pictures and could be classified as CNVs (Copy Number Variation) of uncertain clinical significance.

Conversely, in the third case (patient P7), NGS detected the heterozygous c.2903C>A variant in the NOTCH3 gene. At the protein level, the change determines the introduction of the premature stop codon p.Ser968Ter. The nonsense variant, segregating from the father, was not present in the database of allele frequencies of the general population (gnomAD) and was not still reported in the scientific literature. However, considering its role in the functionality of the protein, it can be classified according to the American College of Medical Genetics and Genomics (ACMG) guidelines as a pathogenetic variant (class 5).

In the fourth case (patient P10), NGS-based gene panel tests revealed a heterozygous c.4855C>A variant in the NOTCH4 gene, determining the amino acid exchange p.Leu1619Met (rs755664291). The missense variant, segregating from the mother, had a frequency allelic equal to 0.000004113 in the general population (gnomAD) and was not reported in scientific literature. It can be classified according to ACMG guidelines as a variant of uncertain significance (VUS) (class 3).

## 4. Discussion

In this paper, we evaluated neurodevelopmental and genetic findings in eleven infants with cerebral AVSs (nine with a VGAM and two with an AVM of the posterior cranial fossa). Of them, only one patient did not undergo endovascular treatment in the first three months of life because of good general conditions. This implies that embolization plays an important role in managing cerebral AVSs: however, beyond the good success rate of the procedure, the neurodevelopmental outcome can be affected by the size of the malformation and the systemic consequences of prolonged heart failure. Recently, Savage et al. performed an individual-participant meta-analysis to identify risk factors associated with all-cause mortality and clinical outcome after VGAM endovascular embolization, including 307 subjects from different small samples (12). They found that a good clinical outcome was achieved in 68% of them. Furthermore, first embolization as a neonate [OR = 6.93; 95% CI (1.99–24.08)] and incomplete embolization [OR = 10.87; 95% CI (1.86–63.55)] were significantly associated with mortality, whereas first embolization as a neonate [OR = 3.24; 95% CI (1.47–7.15)], incomplete embolization [OR = 5.26; 95% CI (2.06–13.43)], and heart failure at presentation [OR = 3.10; 95% CI (1.03–9.33)] were also significantly associated with poor clinical outcomes (12). We confirm this trend: in our cohort, the infants who required more than an embolization had a poor outcome (P1 with a low score in fine and gross motor skills, P4 died, P5 had hydrocephalus and brain atrophy, P7 had focal seizures). Concerning the patients who required inotropes because of heart failure, three died (P4, P8 and P9) and six survived (P1, P3, P5, P6, P7 and P11); among these survivors, one was the patient with low scores in fine and gross motor skills already at 6 months evaluation (P1).

We found diffuse and focal seizures just in one infant who underwent EEG during the NICU stay (P4), while a further infant had focal seizures during follow-up at about three months of life (P7), probably also related to a previous hemorrhagic event. Recorded seizures were mainly electrographic-only type, which is known to be very common in critically ill neonates with many kinds of acute encephalopathy. This finding makes amplitude EEG or continuous video-EEG monitoring essential to ensure good diagnostic accuracy (8). Furthermore, there is increasing evidence suggesting that monitoring and early seizure treatment for neonates with intracranial AVSs could lead to improved neurodevelopmental outcomes (13).

In the group of patients evaluated with evoked potentials, BAEPs and VEPs showed low evidence of sensibility to identify patients with neurological impairment in short-term follow-up. However, patients with MN-SEPs alteration may be associated with possible motor delay during short-term follow-up and may be helpful in scoring patients needed for long-term follow-up in motor development. Somatosensory deficits in post-asphyxia Cerebral Palsy evaluated with MN-SEPs are well known in literature: Suppiej et al. demonstrated that bilaterally absent N20 predicts moderate/severe MRI pattern of injury in neonatal hypoxic-ischemic encephalopathy (9). Similarly, in our small cohort of infants with cerebral AVSs, the patient with bilaterally absent N20 had a low score in fine and gross motor skills at follow-up evaluation. The impairment of motor skills is mainly explained by the fact that the somatosensory system is intimately related to the motor system. Therefore, somatosensory deficits are likely to explain and/or exacerbate motor impairments partially, leading some authors to consider motor involvement in Cerebral Palsy as a sensorimotor disorder (14).

In our cohort, only one patient (1/5, 20%) displayed a moderate developmental delay in the motor area assessed through Bayley-III Scales, whereas none of these patients displayed significant challenges in cognitive and language functioning during the study period. Our findings support the observation that early neurodevelopmental outcomes for neonates treated with embolization procedures are generally favorable, albeit mortality rates for intracranial AVSs presenting in the neonatal period remain high (three patients—27.3%—died during NICU stay). Our study highlights the importance of prompt recognition of cognitive, motor, or language dysfunction in order to drive early intervention in infancy.

Although it is important to report the result of the developmental evaluation at 6 months, it is necessary to remember that these are only preliminary findings since our follow-up is still ongoing, and a specific impairment could be later observed (15), especially in the case of monolaterally impaired MN-SEPs or EEG anomalies. Indeed, patient P7, who initially had N20 absent monolaterally, developed cerebral palsy. These patients need to be strictly followed up to early intercept any deviation from normal development and promptly intervene.

To date, little is known about the genetic background of neonatal intracranial AVSs, and few studies are available. Variants in genes involved in the pathogenesis of vascular anomalies have been identified in single patients in previous studies, including RASA1, EPHB4, and ACVRL1 genes (16, 17). Genetic heterogeneity has been hypothesized for AVSs, with the involvement of “de novo” variants or incomplete penetrance of rare inherited variants. Seventy-two percent of the present patients have been molecularly studied for chromosomal/microchromosomal anomalies and for variants in genes known to be causally related to vascular anomalies.

A novel pathogenetic variant has been detected in one patient only, carrying a variant in the NOTCH3 gene, although the causative role for the AVS is debatable since the gene is related to a progressive cerebral arteriopathy generally manifesting in adulthood (18). The NOTCH3 gene, mapping on 19p13.12, encodes a transmembrane protein that activates several transcriptional factors crucial for embryonic development. Expression studies demonstrated that NOTCH3 can regulate arteriovenous differentiation because is highly expressed in vascular smooth cells (19).

According to animal models of vasculogenesis, its signaling pathway seems to play a role in the etiology of vascular malformations (20). In particular, Hill-Felberg et al. demonstrated that aberrant NOTCH3 signaling may have a role in the pathogenesis of cerebral arteriovenous malformations (20). Its major role is to repress venous differentiation within developing arteries, with the development of arteriovenous shunt if this pathway is impaired (21), such as in the case of an inherited nonsense variant. Although no previous studies have elucidated a causative link between the Notch signaling pathway and AVSs, and our was the first case of NOTCH3 mutation in a patient with choroidal VGAM, we can hypothesize that NOTCH3 may have a role in vascular degeneration typical of cerebral AVSs.

A further additional genomic defect found in one of our patients is of uncertain pathogenicity, consisting of a variant of uncertain significance in the NOTCH4 gene. While NOTCH3 signaling in regulating vascular smooth muscle cell differentiation has been widely reported (22), a possible role of NOTCH4 in contributing to the pathogenesis of brain arteriovenous malformations has been described only in mice (23). Little is still known about the role of NOTCH4 in vascular remodeling (24), and further functional studies are needed to be performed to demonstrate the functional role of this gene in AVS pathogenesis (25). Indeed, NOTCH4 seems to regulate endothelial cell function, considering that impaired NOTCH4 signaling resulted in dilated vessels with evidence of compromised vessel-wall integrity and embryonic lethality in mice (26).

Also, we identified two further variants of uncertain significance, but they do not correspond to a specific disease and do not include vascular genes, at current knowledge.

Furthermore, in our cohort, all females died: further work is needed to explore this suggestion which cannot be explained with only three female cases.

Although based on a small, cross-sectional case series, our eleven patients contribute to better characterizing the neurodevelopmental picture of infants with cerebral AVSs and thus better counseling families about the best therapeutic and follow-up attitudes. We believe that patients with cerebral AVSs should undergo regular neurodevelopmental monitoring and screening to early identify potential deficits, set up an early intervention for high-risk infants, and potentially improve outcomes.

Considering the rarity of the disease, future multicenter studies that systematically will evaluate the cognitive, behavioural, and motor profiles in an objective, quantitative, prospective manner along with the natural history of cerebral AVSs are needed in order to identify potential clinical biomarkers and outcomes measures for testing future novel therapeutic interventions.

## 5. Conclusion

To the best of our knowledge, this is the first study reporting neurophysiological features in infants with cerebral AVSs. With the limitations of the small number of patients evaluated with multimodal neurophysiological tests and the short-term follow-up, we propose MN-SEPs as an ancillary test to discern the most vulnerable infants at the bedside, particularly to identify possible future motor impairment in follow-up. The early identification of a cognitive or motor delay is critical to promptly intervene with personalized rehabilitation treatment and minimize future impairment.

Furthermore, our study also confirms the relevance of continuous EEG monitoring in order to record seizures as early as possible and treat them appropriately. Further studies are needed to better define the timing of EEG recording (preoperative period or postoperative period after intravascular embolization).

The genetic results that we have obtained in two patients are novel and interesting. Conversely, the burden of other variants' interpretation is considerable and difficult. Our next step will be a multicentre study to involve a larger cohort of patients with intracranial AVSs and further investigate genotype background through innovative NGS techniques.

## Data Availability

The original contributions presented in the study are included in the article/Supplementary Material, further inquiries can be directed to the corresponding author.
